# Phosphorylation of HIV-1 Tat by CDK2 in HIV-1 transcription

**DOI:** 10.1186/1742-4690-3-78

**Published:** 2006-11-03

**Authors:** Tatyana Ammosova, Reem Berro, Marina Jerebtsova, Angela Jackson, Sharroya Charles, Zachary Klase, William Southerland, Victor R Gordeuk, Fatah Kashanchi, Sergei Nekhai

**Affiliations:** 1 Center for Sickle Cell Disease, Howard University College of Medicine, 520 W Street N.W., Washington, DC 20059, USA; 2 Department of Biochemistry and Molecular Biology, Howard University College of Medicine, 520 W Street N.W., Washington, DC 20059, USA; 3 Program in Genetics, Howard University College of Medicine, 520 W Street N.W., Washington, DC 20059, USA; 4 Department of Biochemistry and Molecular Biology, The George Washington University Medical Center, 2300 I Street N.W., Washington, DC 20037, USA; 5 Children's National Medical Center, CRI Center III, 111 Michigan Ave., N.W. Washington, D.C. 20010-2970, USA

## Abstract

**Background:**

Transcription of HIV-1 genes is activated by HIV-1 Tat protein, which induces phosphorylation of RNA polymerase II (RNAPII) C-terminal domain (CTD) by CDK9/cyclin T1. Earlier we showed that CDK2/cyclin E phosphorylates HIV-1 Tat *in vitro*. We also showed that CDK2 induces HIV-1 transcription *in vitro *and that inhibition of CDK2 expression by RNA interference inhibits HIV-1 transcription and viral replication in cultured cells. In the present study, we analyzed whether Tat is phosphorylated in cultured cells by CDK2 and whether Tat phosphorylation has a regulatory effect on HIV-1 transcription.

**Results:**

We analyzed HIV-1 Tat phosphorylation by CDK2 *in vitro *and identified Ser^16 ^and Ser^46 ^residues of Tat as potential phosphorylation sites. Tat was phosphorylated in HeLa cells infected with Tat-expressing adenovirus and metabolically labeled with ^32^P. CDK2-specific siRNA reduced the amount and the activity of cellular CDK2 and significantly decreased phosphorylation of Tat. Tat co-migrated with CDK2 on glycerol gradient and co-immunoprecipitated with CDK2 from the cellular extracts. Tat was phosphorylated on serine residues *in vivo*, and mutations of Ser^16 ^and Ser^46 ^residues of Tat reduced Tat phosphorylation *in vivo*. Mutation of Ser^16 ^and Ser^46 ^residues of Tat reduced HIV-1 transcription in transiently transfected cells. The mutations of Tat also inhibited HIV-1 viral replication and Tat phosphorylation in the context of the integrated HIV-1 provirus. Analysis of physiological importance of the S^16^QP(K/R)^19 ^and S^46^YGR^49 ^sequences of Tat showed that Ser^16 ^and Ser^46 ^and R^49 ^residues are highly conserved whereas mutation of the (K/R)^19 ^residue correlated with non-progression of HIV-1 disease.

**Conclusion:**

Our results indicate for the first time that Tat is phosphorylated *in vivo*; Tat phosphorylation is likely to be mediated by CDK2; and phosphorylation of Tat is important for HIV-1 transcription.

## Background

The human immunodeficiency virus type 1 (HIV-1) requires host cell factors for all steps of the viral replication [1,2]. Recently, multiple covalent modifications of viral proteins that regulate virus-host protein interactions have been described, such as phosphorylation, acetylation and ubiquitination. Phosphorylation has been reported for almost all HIV-1 accessory proteins, including Vpu [3], Vpr [4], Vif [5], Nef [6], and Rev [7]. Transcription of HIV-1 viral genes is induced by a viral transactivator protein (Tat) [1,2]. The activation domain of Tat (amino acids 1–48) interacts with host cell factors, whereas the positively charged RNA-binding domain (amino acids 49–57) interacts with HIV-1 transactivation response (TAR) RNA [1,2]. In cell-free transcription assays Tat induces exclusively elongation of transcription [8,9]. *In vivo*, Tat additionally induces initiation of transcription from the integrated HIV-1 promoter [10-12]. Tat stimulates formation of transcription complex containing TATA-box-binding protein (TBP) but not TBP-associated factors (TAFs), thus indicating that Tat may enhance initiation of transcription [10], apparently in agreement with the earlier observation that Tat binds directly to the TBP-containing basal transcription factor TFIID [13]. Tat activates HIV-1 transcription by recruiting transcriptional co-activators that include Positive Transcription Elongation Factor b (P-TEFb), containing CDK9/cyclin T1; an RNA polymerase II C-terminal domain kinase [9,14,15] and histone acetyl transferases [16-18]. Whereas P-TEFb induces HIV-1 transcription from non-integrated HIV-1 template [9,14,15], histone acetyl transferases allow induction of integrated HIV-1 provirus [16-18]. Additional CTD kinases, including CDK2 and CDK7 were also reported to be activated by Tat and to induce functional CTD phosphorylation [19,20]. Tat itself is a subject for covalent modifications by host cell proteins. Tat is directly acetylated at lysine 28, within the activation domain, and lysine 50, in the TAR RNA binding domain [21]. Tat is also ubiquitinated at lysine 71 and its ubiquitination stimulates the transcriptional properties of Tat [22]. Recently, Tat was shown to be methylated by the arginine methyltransferase, PRMT6 and the arginine methylation of Tat negatively regulated its transcriptional activity [23]. Surprisingly, in spite of the interaction of Tat with P-TEFb and probably other kinases and its involvement in multiple protein phosphorylation reactions, the phosphorylation of HIV-1 Tat has only been reported *in vitro *[24], but not *in vivo *[25]. HIV-2 Tat was reported to be phosphorylated *in vivo *presumably by CDK9, but this phosphorylation was not important for Tat-2 function as a transcriptional activator [26]. We previously reported that Tat dynamically interacts with CDK2/cyclin E and is also phosphorylated by CDK2/cyclin E *in vitro *[20]. This dynamic interaction greatly stimulated the activity of CDK2/cyclin E toward phosphorylation of CTD *in vitro *[20]. In the present study we investigated whether Tat is phosphorylated *in vivo *and whether this phosphorylation has a regulatory role in Tat-activated HIV-1 transcription.

## Results

### Tat is phosphorylated by CDK2 *in vitro *and Ser-16 and Ser-46 residues of Tat are potential phosphorylation sites

**Figure 1:**
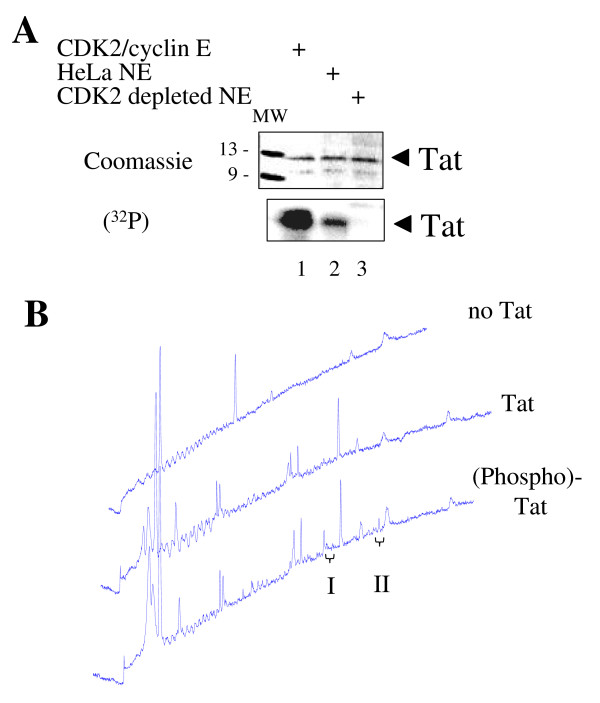
**Analysis of HIV-1 Tat phosphorylated by CDK2/cyclin E *in vitro***. ***A*, Tat is phosphorylated by CDK2**. Recombinant Tat was phosphorylated *in vitro *by purified CDK2/cyclin E (lane 1), by HeLa nuclear extract (lane 2) or by CDK2-depleted HeLa nuclear extract (lane 3). Tat was resolved by 12% SDS Tris-Tricin PAGE. The gel was stained with Coomassie blue (upper panel) and exposed to Phospho Imager screen (lower panel). ***B*, HPLC profiles of Tat peptides after trypsin cleavage**. Recombinant Tat was phosphorylated *in vitro *by purified CDK2/cyclin E, resolved by 12% SDS Tris-Tricin PAGE, and subjected to in-gel trypsin digestion. The eluted peptides were resolved by reverse phase chromatography on μRPC C2/C18 ST 4.6/100 column. *No Tat*, mock trypsin digest without Tat. *Tat*, digest of non-phosphorylated Tat. *(Phospho)-Tat*, digestion of phosphorylated Tat. I and II, peaks identified in the elution profile of phosphorylated Tat that were subjected to MALDI TOF/TOF mass spectrometry.

We previously showed that Tat is phosphorylated by recombinant CDK2/cyclin E *in vitro *and that Tat's Ser16 was a potential phosphorylation site [20]. Indeed recombinant CDK2/cyclin E efficiently phosphorylates Tat (Fig. 1A, lane 1). Tat can also be phosphorylated by HeLa nuclear extract (Fig. 1A, lane 2). Immunodepletion of CDK2 from HeLa nuclear extract completely abolished Tat phosphorylation (Fig. 1A, lane 3) suggesting that under these conditions Tat was largely phosphorylated by CDK2. Upon analysis of the sequence of Tat, the (S/T)_0_P_1_K_2_(K/R)_3 _consensus motif for serine phosphorylation by CDK2 [27,28] was not found, but several sequences were found that partially matched this motif: S^16^QP(K/R)^19 ^, S^46^YGR^49 ^, S^68^LSK^71 ^. To determine potential phosphorylation sites, Tat phosphorylated *in vitro *was analyzed by Mass spectrometry. For this purpose, purified recombinant Tat was phosphorylated with recombinant CDK2/cyclin E *in vitro *followed by immunoprecipitation, SDS-PAGE purification, in-gel digestion with trypsin, and HPLC purification. Analysis of HPLC eluates showed presence of two peaks in the digest generated from phosphorylated Tat that were absent in digest of non-phosphorylated Tat (Fig. 1B). These two peaks were collected and subjected to MALDI-TOF mass spectrometry. Masses of the peptides over 900 Da were acquired and analyzed with FindPep tool [29] using the sequence of Tat (MEPVDPNLEPWKHPGSQPRTACNNCYCKKCCFHCYACFTRKGLGISYGRKKRRQRRRAPQDSQTHQASLSKQ) as input. The masses of peptides that did not match to Tat were further compared with Tat peptides from which we subtracted 18 Da, assuming β-elimination of phosphoric acid which is likely to occur during MALDI-TOF analysis [30]. Matched peptides shown in Table 1, indicate that Peak I contains peptides with Serine 46 as a potential phosphorylation site, whereas Peak II contains peptides with Serine 16 as a potential phosphorylation site. All matched peptides contain internal lysine or arginine residues, and thus they are apparently products of incomplete digestion by trypsin, which could be a result of incomplete in-gel digestion. Also acquisition of peptide with masses over 900 Da would only allow detection of relatively large peptides. The data suggest that Tat is phosphorylated *in vitro *by CDK2 and that this phosphorylation might take place at Ser^16 ^or Ser^46 ^residues within S^16^QPR^19 ^and S^46^YGR^49 ^sequences that partially match to the (S/T)P×(K/R) consensus sequence for CDK2 phosphorylation [28].

**Table 1:** Determination of sites of phosphorylation in Tat

**Peak**	**Peptides matching to Tat**	**MW**	-18Da	Matching peptides
**I**	(T)RKGLGISYG(R)	950.606	932.606	932.606 931.553
	(L)GISYGRKKRR/(Q)	1220.7	1202.7	1202.7 1202.698
	(S)YGRKKRRQRR/(R)	1403.779	1385.779	1385.779 1385.777

**II**	(L)EPWKHPGSQPRTACNNCYCK/(K)	2319.272	2301.272	2301.272 2301.274
	(P)WKHPGSQPRTAC(N)	1367.563	1349.563	1349.563 1349.544 1349.563 1349.544
	(P)WKHPGSQPRTACNN(C)	1595.811	1577.811	1577.811, 1576.779
	(P)NLEPWKHPG(S)	1077.606	1059.606	1059.606

### Tat is phosphorylated in cultured cells

**Figure 2:**
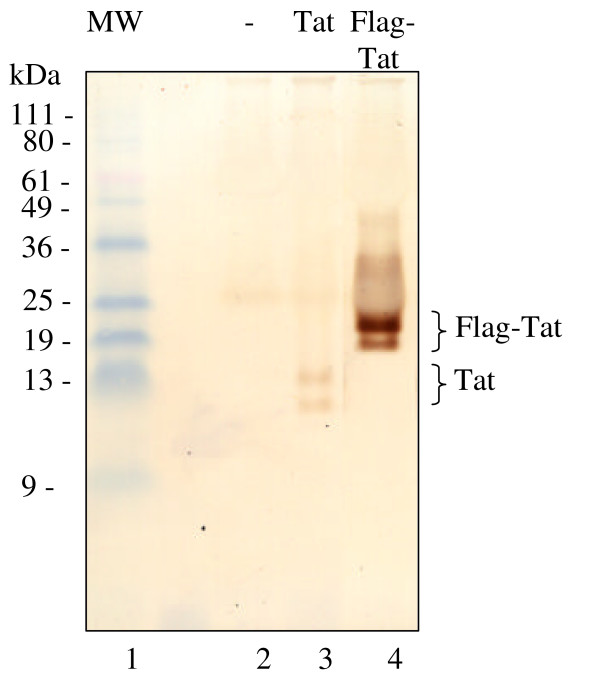
**Expression of untagged and Flag-tagged Tat**. COS-7 cells were transfected with Tat (lane 3) and Flag-tagged Tat (lane 4) expression vectors or mock-transfected (lane 2). At 48 hours post transfection cells were lysed and Tat was immediately immunoprecipitated with anti-Tat rabbit polyclonal antibodies (lanes 2–4). Immunoprecipitated Tat was resolved by 15% Tris-Tricine SDS-PAGE, transferred to polyvinylidene fluoride membrane and immunoblotted with anti-Tat monoclonal antibodies using the 3,3'-diaminobenzidine enhancer system. Positions of Tat and Flag-Tat are indicated. Lane 1, prestained 10 kDa molecular weight markers.

**Figure 3:**
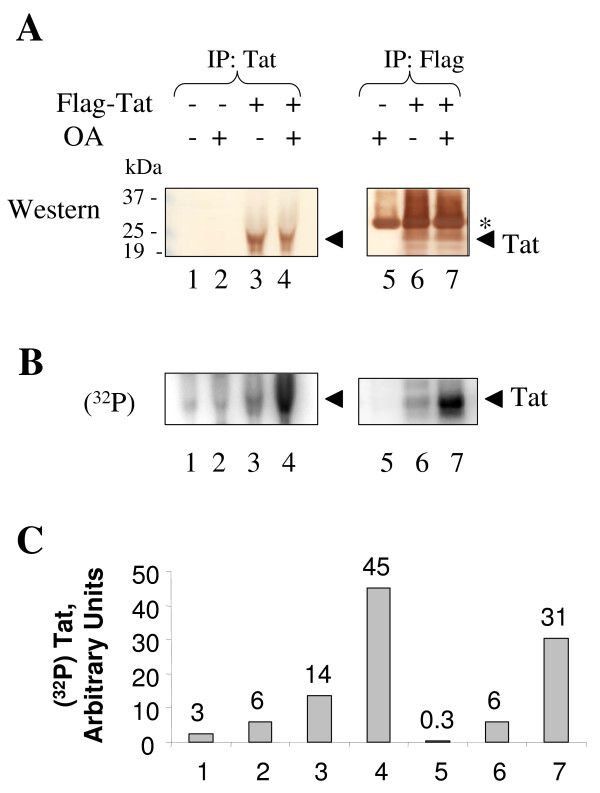
**HIV-1 Tat is phosphorylated in cultured cells**. HeLa cells were infected with recombinant adenovirus expressing Flag-tagged Tat as described in Methods (lanes 3, 4, 6 and 7). Lanes 1, 2, and 5 – control uninfected cells. At 48 hours post infection cells were labeled with (^32^P)-orthophosphate for 2 hours without (lanes 1, 3 and 6) or with (lanes 2, 4, 5 and 7) 1 μM okadaic acid (OA). Whole cell extracts were prepared and Tat was immunoprecipitated with anti-Tat rabbit polyclonal antibodies (lanes 1–4) or anti-Flag monoclonal murine antibodies (lanes 5–7). Immunoprecipitated Tat was resolved by 15% Tris-Tricine SDS-PAGE, and transferred to polyvinylidene fluoride membrane. *A*, immunoblot of the membrane with anti-Tat monoclonal antibodies using the 3,3'-diaminobenzidine enhancer system. *B*, autoradiography of the membrane on Phosphor Imager. *C*, quantification of panel B. The position of light chain of IgG recognized in anti-Flag immunoprecipitates by anti-mouse HRP-conjugated secondary antibodies is indicated by asterisk.

Previous attempts to detect Tat phosphorylation *in vivo *were unsuccessful [25]. We hypothesized that low level of Tat expression after transfection and/or rapid de-phosphorylation by cellular phosphatases might prevent detection of Tat phosphorylation *in vivo*. To overcome these difficulties, we expressed Flag-tagged Tat which we found to be expressed to a higher level in COS-7 cells than untagged Tat (Fig. 2, compare lanes 3 and 4). To facilitate expression of Flag-Tat in HeLa cells we used adenovirus-mediated expression of Tat [31]. HeLa cells were infected with Adeno-Tat and incubated 48 hours post infection to allow expression of Tat. Then cells were pulsed with (^32^P)-labeled orthophosphate, and Tat was immunoprecipitated from cellular lysates using monoclonal anti-Flag or polyclonal anti-Tat antibodies. Immunoprecipitated proteins were resolved by SDS-PAGE on 15% Tris-Tricine gel [32] and transferred to PVDF membrane. The membrane was probed with monoclonal anti-Tat antibodies (Fig. 3A) using 3,3'-Diaminobenzidine enhancer system (DABM, Sigma), and also exposed to a PhosphoImager screen (Fig. 3B). Both antibodies precipitated a well detectable amount of Tat protein (Fig. 3A, lanes 3, 4, 6 and 7). Under these experimental conditions, Tat was phosphorylated (Figs. 3B and 3C, compare lane 3 to lane 1 and lane 6 to lane 5). Next we treated cells with okadaic acid, an inhibitor of PPP-type phosphatases, to prevent rapid dephosphorylation of Tat in the cells and during the lysis procedure. Treatment with okadaic acid did not change the amount of precipitated Tat (Fig. 3A, lanes 3, 4, 6 and 7). In contrast, Tat phosphorylation was significantly enhanced when cells were treated with okadaic acid (Figs.3B and 3C, lanes 4 and 7). Taken together, these results indicate that Tat is phosphorylated *in vivo *and that Tat phosphorylation is enhanced when cells are treated with the inhibitor of PPP-phosphatases.

### CDK2 phosphorylates Tat in cultured cells

**Figure 4:**
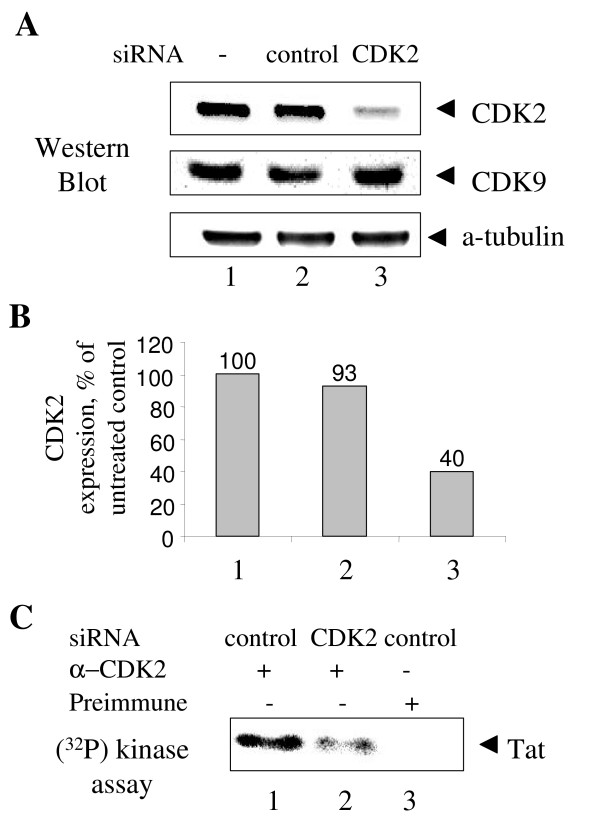
**CDK2-directed siRNA inhibits CDK2 expression**. ***A***, CDK2-directed siRNA inhibits expression of CDK2. HeLa cells were transfected with siRNAs targeting CDK2 (lane 3) or non-targeting control pool (control, lane 2). Lane 1, untransfected cells. At 48 hours post-transfection cells were lysed and cellular extracts were resolved on 12% Tris-Tricine SDS-PAGE and analyzed by immunoblotting analysis with antibodies against CDK2, CDK9 or α-tubulin. *B*, quantification of the CDK2 expression in panel A using α-tubulin expression level for normalization. *C*, CDK2-directed siRNA inhibits enzymatic activity of CDK2. CDK2 was precipitated from cellular extracts prepared from HeLa cells transfected with siRNAs targeting CDK2 (lane 2) or non-targeting control (lanes 1 and 3). Lanes 1 and 2, precipitation with rabbit anti-CDK2 antibodies. Lane 3, precipitation with rabbit preimmune serum. Immunoprecipitates were incubated with γ-(^32^P)ATP and recombinant Tat (see Methods), resolved on 12% Tris-Tricine SDS-PAGE and analyzed by autoradiography on Phosphor Imager. Position of Tat is indicated.

**Figure 5:**
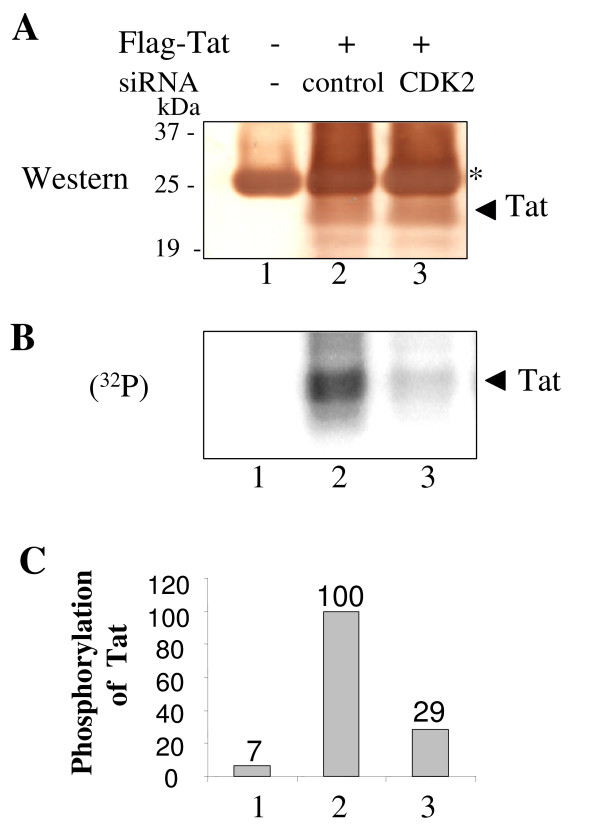
**CDK2-directed siRNA blocks Tat phosphorylation**. *A*, HeLa cells were infected with Adeno-Tat (lanes 2 and 3). At 4 hours post infection, cells were transfected with siRNAs targeting CDK2 (lane 3) or non-targeting control pool (lane 2). Lane 1 – control cells. At 48 hours post-infection cells were labeled with (^32^P)-orthophosphate for 2 hours. Whole cell extract was subjected to immunoprecipitation with anti-Flag antibodies, resolved by 15% Tris-Tricine SDS-PAGE, and transferred to polyvinylidene fluoride membrane. *A*, immunoblot of the membrane with anti-Tat monoclonal antibodies using the 3,3'-diaminobenzidine enhancer system. *B*, autoradiography of the membrane on Phosphor Imager screen. *C*, quantification of the panel B. Position of Tat is indicated by arrow. The position of light chain of IgG recognized in anti-Flag immunoprecipitates by anti-mouse HRP-conjugated secondary antibodies is indicated by asterisk.

**Figure 6:**
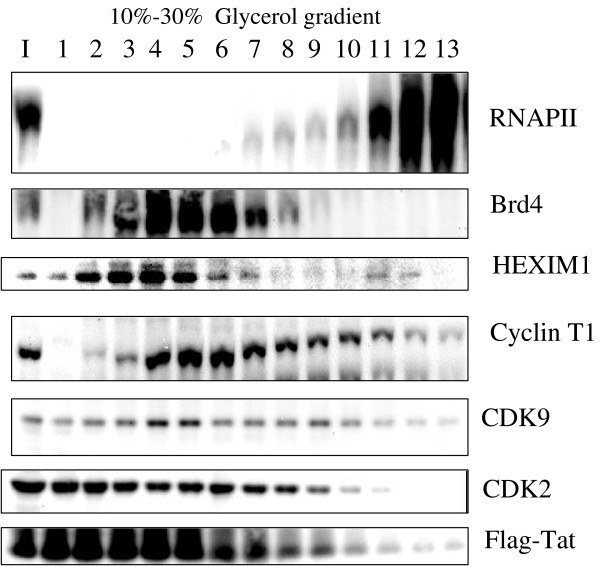
**Tat and CDK2 co-migrate on glycerol gradient**. 293T cell lysated from the cells infected with Adeno-Tat were fractionated on 10%–30% glycerol gradients by centrifugation and analyzed with indicated antibodies by Immunoblotting.

**Figure 7:**
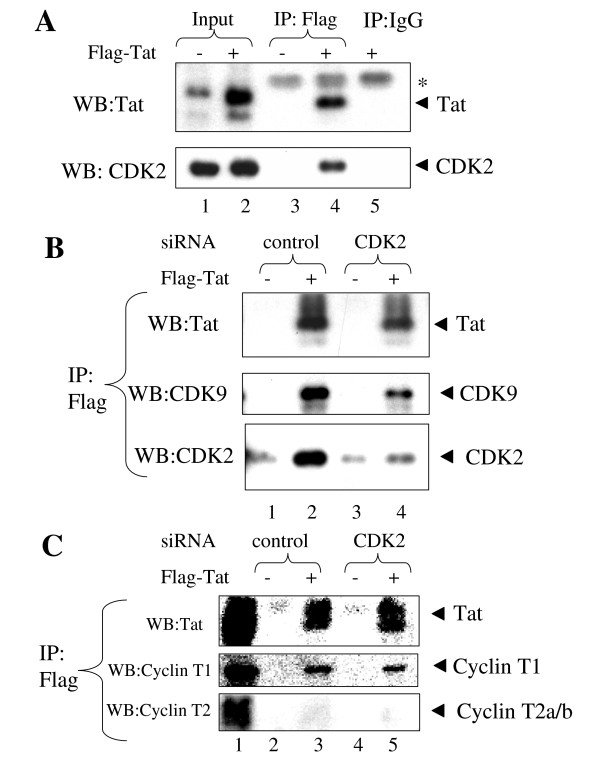
***A*, CDK2 associates with Tat in cultured cells**. Flag-tagged Tat was expressed in HeLa cells by Adeno-Tat infection and precipitated with anti-Flag antibodies from cellular lysate. Co-precipitated proteins were resolved on 12% Tris-Tricine PAGE and analyzed by immunoblotting analysis with anti-CDK2 and anti-Tat antibodies. Lane 1, input control without Tat. Lane 2, input control with Tat. Lanes 3 and 4, extract without or with Tat precipitated with anti-Flag antibodies. Lane 5, extract with Tat precipitated with preimmune mouse IgG. The position of light chain of IgG recognized in anti-Flag immunoprecipitates by anti-mouse HRP-conjugated secondary antibodies is indicated by asterisk. ***B*, CDK2-specific siRNA inhibits association of Tat with CDK2**. Flag-tagged Tat was expressed in 293T cells by transfection (lanes 2 and 4). Cells were transfected with non-targeting (lanes 1 and 2) or CDK2-specific (lanes 3 and 4) siRNAs. Lysates were precipitated with anti-Flag antibodies, resolved on 12% Tris-Tricine PAGE and immunoblotted with anti-Tat, anti-CDK9 or anti-CDK2 antibodies. ***C*, CDK2-specific siRNA does not affect association of Tat with cyclin T1**. Flag-tagged Tat was expressed in 293T cells by transfection (lanes 3 and 5). Cells were transfected with non-targeting (lanes 2 and 3) or CDK2-specific (lanes 4 and 5) siRNAs. Lysates were precipitated with anti-Flag antibodies, resolved on 12% Tris-Tricine PAGE and immunoblotted with anti-Tat, anti-CDK9 or anti-CDK2 antibodies. Lane 1, input.

We next investigated whether Tat phosphorylation was mediated by CDK2 *in vivo *using CDK2-directed RNA interference [33]. HeLa cells were infected with Adeno-Tat and subsequently transfected with siRNAs against CDK2. Transfection of HeLa cells with siRNAs against CDK2 decreased the level of expression of CDK2 by 2.5-fold (Figs. 4A and 4B, lane 3). A control non-targeting siRNA pool did not affect expression of CDK2 (Figs. 4A and 4B, lane 2). The non-targeting siRNA control was used to ensure that transfection itself did not affect CDK2 expression. Western blot analysis of tubulin and CDK9 was used as control for the specificity of siRNAs. As shown in Fig. 4A transfection with both siRNAs did not affect the level of α-tubulin expression. To ensure that CDK2-directed siRNA decreased the enzymatic activity of cellular CDK2, CDK2 was immunoprecipitated from cells transfected with non-targeting or CDK2-directed siRNA and assayed for its enzymatic activity using recombinant Tat as a substrate. The activity of CDK2 was decreased in the cells transfected with CDK2-directed siRNA (Fig. 4C, lane 2) as compared to the cells transfected with non-targeting siRNA (Fig. 4C, lane 1). Next the cells were infected with Adeno-Tat, transfected with non-targeting or CDK2-directed siRNAs and pulse-labeled with (^32^P). In this experiment no okadaic acid was used. Inhibition of CDK2 by siRNA reduced the level of Tat phosphorylation by 3-fold (Figs. 5A,B and 5C, compare lanes 2 and 3). Thus CDK2 is likely to mediate Tat phosphorylation in cultured cells. To analyze whether Tat and CDK2 might be present in the same molecular weight complex, we analyzed sedimentation of Tat and CDK2 by ultracentrifugation on a glycerol gradient (Fig. 6). Both Tat and CDK2 co-migrated in fractions 1–8 (Fig. 6). The CDK9 was present in most of the fractions with the peak in fractions 4–5 and 9–10 (Fig. 6), which is likely to correspond to low and high molecular weight P-TEFb complexes. HEXIM1, and Brd4 were mostly present in fractions 2–6, although HEXIM1 was also detected in higher molecular weight fractions 10 and 11 (Fig. 6). Neither Tat nor CDK2 co-migrated with RNAPII which was present in fractions 1–13 (Fig. 6). We further analyzed association of Tat with CDK2 by immunoprecipitation. Flag-Tat was expressed in HeLa cells by infection with Adeno-Tat and precipitated from cellular extracts with anti-Flag antibodies (Fig. 7A). CDK2 co-precipitated with Tat (Fig. 7A, lane 4). CDK2 was not precipitated with anti-Tat antiserum from non-infected cells (Fig. 7A, lane 3) or not with non-specific preimmune serum from adeno-Tat infected cells (Fig. 7A, lane 5). Inhibition of CDK2 expression by CDK2-specific RNAi significantly reduced CDK2 co-precipitated to Tat apparently due reduction of the expressed CDK2 (Fig. 7B, compare lane 4 to lane 2). Association of Tat with CDK9 was slightly reduced (Fig. 6B) but this reduction correlated to the decreased amount of Tat precipitated by anti-Flag antibodies. Binding of Tat-cyclin T1 was not reduced (Fig. 7C, lanes 3 and 5). The cyclin T2 did not precipitate with Tat (Fig. 7C), which indicated a specificity of the immunoprecipitation. Taking together, these results suggest that CDK2 associates with Tat in cultured cells, and that inhibition of CDK2 expression prevents Tat phosphorylation. Thus, CDK2 is likely to phosphorylate Tat directly in cultured cells.

### Tat is phosphorylated on serine residues *in vivo*

**Figure 8:**
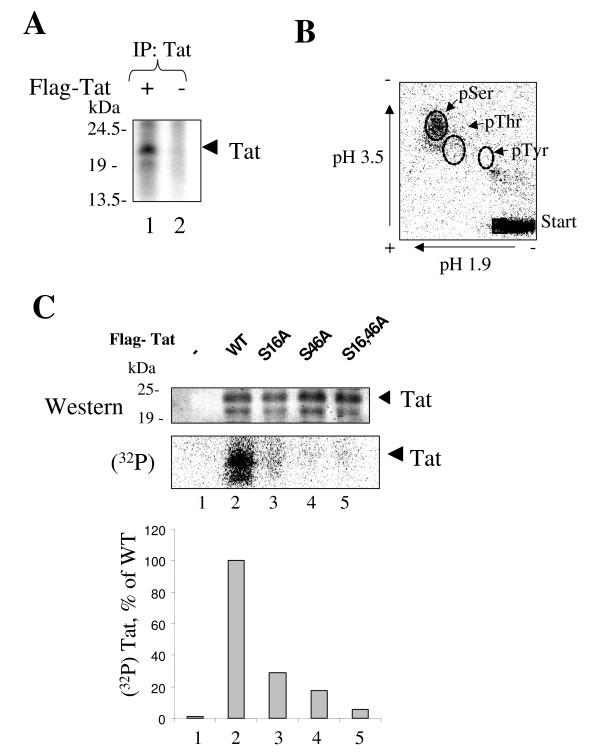
**HIV-1 Tat is phosphorylated on S^16 ^and S^46 ^residues *in vivo***. ***A***, HeLa cells were infected with recombinant adenovirus expressing Flag-tagged Tat as described in *Methods*. At 48 hours post infection cells were labeled with (^32^P)-orthophosphate for 2 hours with 1 μM okadaic acid (OA). Lane1, Flag-Tat was immunoprecipitated from whole cell extracts with anti-Flag antibodies and resolved by 15% Tris-Tricine SDS-PAGE. Lane 2, control mock-transfected cells. The picture is an autoradiogram. ***B***, Tat peptides were eluted from the gel shown in panel A by overnight incubation with trypsin and subjected to acid hydrolysis as described in *Materials and Methods*. The hydrolyzed material was spotted on nitrocellulose plate and examined by two-dimensional thin layer electrophoresis and autoradiography. The indicated positions of non-radioactive phospho-amino acid standards were visualized by staining with 0.5% ninhydrin in ethanol. *C*, Mutations of S^16 ^and S^46 ^reduce Tat phosphorylation *in vivo*. 293T cells were transfected with vectors expressing Flag-tagged WT Tat (lane 2), Tat S16A (lane 3), Tat S46A (lane 4) or Tat S16,46A (lane 5). Lane 1, mock transfection. At 48 hours post-transfection the cells were labeled with (^32^P)-orthophosphate for 2 hours. Whole cell extract was subjected for immunoprecipitation with anti-Flag antibodies, resolved by 15% Tris-Tricine SDS-PAGE, and analyzed by Western blotting with polyclonal anti-Tat antibodies and on Phosphor Imager. Quantification is shown in the lower panel. Position of Tat is indicated by arrow.

We next determined whether serine, threonine or tyrosine residues of Tat were phosphorylated *in vivo*. HeLa cells were infected with Adeno-Tat, labeled with (^32^P), and treated with okadaic acid to achieve a higher level of Tat phosphorylation. Tat was immunoprecipitated with anti-Flag antibody and resolved on 15% SDS Tris-Tricine PAGE (Fig. 8A, lane 1). Phosphoamino acid analysis of radioactive Tat extracted from the gel (see *Materials and Methods*) showed presence of phospho-serines but not phospho-threonines or phospho-tyrosines in (^32^P)-labeled Tat (Fig. 8B). Thus, only serine residues of Tat were phosphorylated *in vivo*.

### Phosphorylation of S^16 ^and S^46 ^residues of Tat *in vivo*

We next investigated a possibility that Tat might be phosphorylated on S^16 ^or S^46 ^residues *in vivo*. We generated mutants of Flag-Tat in which either or both Ser residues were substituted by Ala. 293T cells were transfected with WT and mutant Tat-expressing vectors, Tat was precipitated with anti-Flag antibodies and analyzed on 15% SDS Tris-Tricine PAGE followed by PhosphoImager analysis. Expression of Tat was verified by Western blotting (Fig. 8C). While we could detect phosphorylation of WT Tat (Fig. 8C, lane 2), the Tat S16A mutant and Tat S46A mutant were about 2–3 fold less phosphorylated (Fig. 8C, middle and lower panels, lanes 3 and 4). The Tat S16,46A double mutant was even less phosphorylated (Fig. 8C, lane 5). Our results indicate that both S^16 ^and S^46 ^are likely to be phosphorylated *in vivo*.

### Contribution of S^16 ^and S^46 ^residues of Tat to HIV-1 transcription

**Figure 9:**
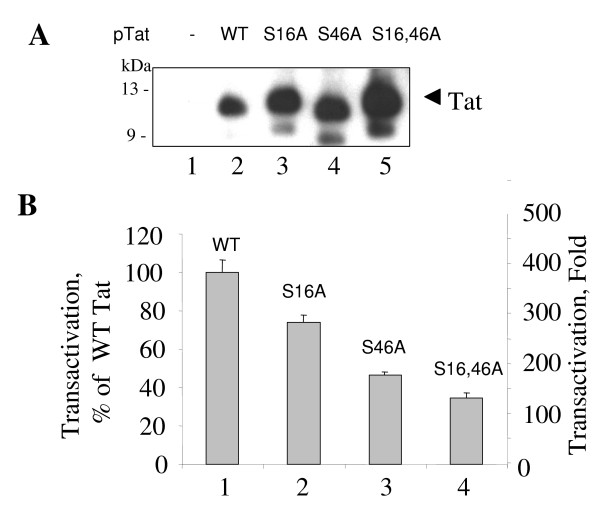
**Mutations of Ser^16 ^and Ser^46 ^of Tat reduce its transactivation potential**. ***A***, COS-7 cells were transfected with WT Tat, Tat S16A, Tat S46A or Tat S16,46A expression vectors. At 48 hours posttransfection, the cells were lysed. Tat was immunoprecipitated from the lysates with rabbit polyclonal antibodies, resolved by 15% Tris-Tricine SDS-PAGE and immunoblotted with monoclonal anti-Tat antibodies. ***B***, HeLa cells were transfected with the HIV-1 LTR-LacZ expression vector alone (not shown here) and in combination with WT Tat, Tat S16A, Tat S46A or Tat S16,46A expression vectors. At 48 hours posttransfection, cells were lysed and analyzed for β-galactosidase activity with ONPG.

We next investigated the functional relevance of Tat's S^16 ^and S^46 ^residues in HIV-1 transcription. We generated mutants of Tat in which either or both Ser residues were substituted by Ala. To ensure expression of the mutants, COS-7 cells were transfected with WT and mutant Tat-expressing vectors and cellular lysates were analyzed on 15% SDS Tris-Tricine PAGE followed by Western blot with anti-Tat antibodies. As shown in Fig. 9A, all Tat mutants were expressed, with the level of expression of Tat mutants higher than the WT Tat. The higher expression level of non-tagged Tat mutants was a reproducible effect and was not a consequence of the difference in the amount of transfected DNA. The effect of Tat mutations on the ability of Tat to activate HIV-1 LTR promoter was analyzed in HeLa cells co-transfected with Tat-expression vectors and HIV-1 LTR-LacZ reporter plasmid (Fig. 9B). Non-mutated Tat (WT) increased the level of transcription by 400-fold (Fig 9B). HIV-1 transcription induction by the Tat S16A mutant was approximately 75% that of WT Tat (Fig. 9B), while transactivation by the Tat S46A mutant was about 2 times lower than with the WT Tat (Fig. 9B) and induction by the double S16, 46A Tat mutant was 3-times lower than that of WT Tat (Fig. 9B). Thus, mutation of either Ser^16 ^or Ser^46 ^of Tat interferes with the level of Tat-transactivation and mutation of both residues has an additive effect.

### Contribution of S^16 ^and S^46 ^residues of Tat to the HIV-1 viral production and Tat phosphorylation in the context of the integrated HIV-1 provirus

**Figure 10:**
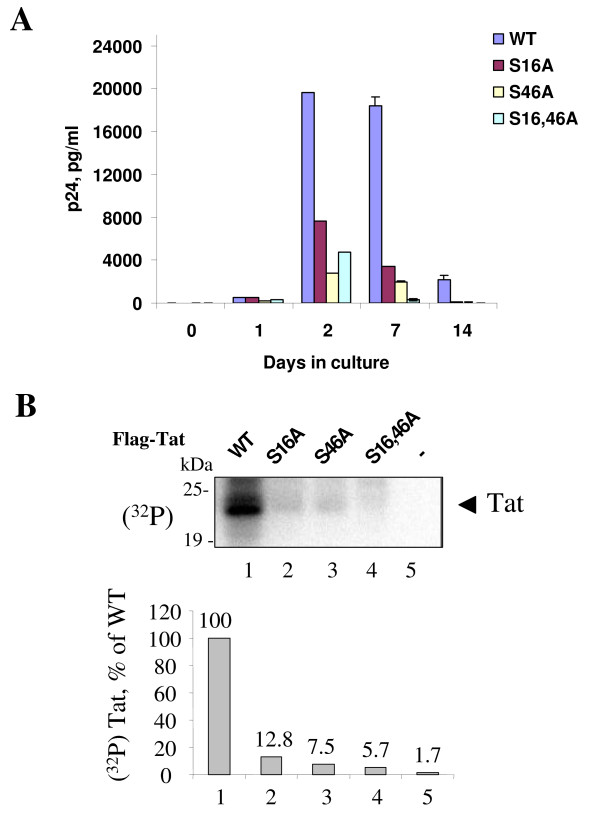
***A*, Mutations of S^16 ^and S^46 ^of Tat reduce its ability to induce viral production**. HLM1 is a HeLa derived cells containing one copy of integrated HIV-1 proviral genome with a Tat-defective mutation. Various Flag-tagged Tat (WT Tat, Tat S16A, Tat S46A or Tat S16,46A) expression vectors were used for HLM-1 transfections. Cells were cultured in complete media in absence of G418 for 14 days. The supernatants were collected at Day 0, 1, 2, 7 and 14, and analyzed for p24 by ELISA assay. ***B*, Mutations of S^16 ^and S^46 ^of Tat inhibit Tat phosphorylation during one round of viral replication**. HLM1 cells were transfected with vectors expressing Flag-tagged Tat (WT Tat, Tat S16A, Tat S46A or Tat S16,46A). At 48 hours post transfection the cells were labeled with (^32^P)-orthophosphate for 2 hours with 1 μM okadaic acid. Flag-Tat was immunoprecipitated from whole cell extracts with anti-Flag antibodies and resolved by 15% Tris-Tricine SDS-PAGE. The gel was dried and exposed to Phosphor Imager screen. Lane 1, Wt Tat. Lane 2, Tat S16A. Lane 3, Tat S46A. Lane 4, Tat S16,46A. Lane 5, mock-transfected cells. The picture is an autoradiogram.

We determined whether mutations of Tat S16A and/or S46A have an effect on the ability of Tat to induce HIV-1 transcription from an integrated HIV-1 provirus. We used HLM-1 cells (AIDS Research and Reference Reagent Program) that were derived from HeLa-CD4+ cells containing an integrated copy of HIV-1 proviral genome with a Tat-defective mutation (termination linker at the first AUG). HLM-1 cells are negative for virus particle production, but can be induced to express high levels of infectious HIV-1 after transfection with Tat. We transfected the HLM-1 cells with wild type or mutant Tat vectors and tested supernatants for the presence of HIV-1 particles using p24 gag antigen ELISA at day 0, day 1, day 2, day 7 and day 14 posttransfection. Neither S16A nor S46A mutants of Tat efficiently induced HIV-1 viral production (Fig. 10A). The double S16, 46A mutant also had a reduced activity (Fig. 10A). To phosphorylate Tat during virus replication, we pulsed HLM-1 cells transfected with WT and mutant Tat with (^32^P) orthophosphate and also treated the cells with okadaic acid. Tat was immunoprecipitated with anti-Flag antibodies, resolved on 15% SDS PAGE and its phosphorylation was detected by PhosphoImager. While WT Tat was phosphorylated, the mutants were not phosphorylated efficiently (Fig. 10B). These data indicate that the S16A and S46A mutations of Tat interfere with the ability of Tat to activate integrated HIV-1 provirus, and prevent Tat phosphorylation during one round of viral replication.

### Correlation of mutations in putative CDK2 recognition sites on Tat with disease progression in HIV infected humans

**Figure 11:**
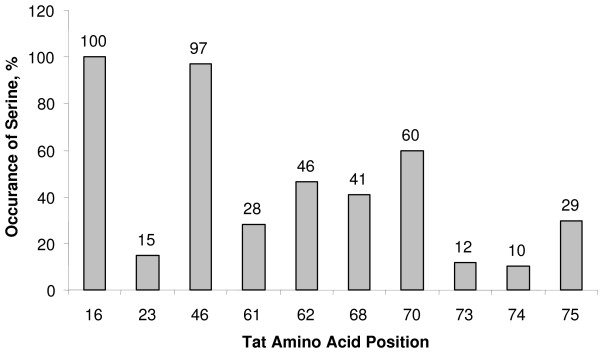
**Serine residues at position 16 and 46 are highly conserved in Tat isolates**. The 158 sequences of Tat isolates deposited to PubMed database were analyzed for the presence of serines at position 16, 23, 46, 61, 68, 70, 73, 74 and 75. Presence of a serine at the indicated position is presented as a percent of the total number of the isolates analyzed.

As we discussed above, analysis of the sequence of Tat for the presence of the (S/T)_0_P_1_K_2_(K/R)_3 _consensus motif for serine phosphorylation by CDK2 [27,28] showed that several sequences partially matched this motif: ^16^SQP(K/R)^19 ^, ^46 ^SYGR^49 ^, ^68^SLSK^71 ^. We determined conservancy of S^16^, S^46 ^and S^68 ^residues. We analyzed 158 sequences of Tat isolates deposited in the PubMed database. Both S^16 ^and S^46 ^were highly conserved with an occurrence of 100% or nearly 100% (Fig. 11). In contrast, S^68 ^was present in 41%, and S^70 ^– in 60% of the Tat isolates and all other serines were present in less than 50% (Fig. 11). This analysis indicates that S^16 ^and S^46 ^residues might be critical for HIV-1 replication. Because S^16 ^and S^46 ^residues were 100% conserved, it was not possible to analyze the effect of the mutation of these residues on the HIV-1 progression. We took advantage of the notion that a lysine or arginine present in the fourth position of the (S/T)_0_P_1_K_2_(K/R)_3 _consensus motif is critical for the recognition of the motif by CDK2 [27,28]. Thus we analyzed whether (K/R)^19 ^and R^49 ^residues within ^16^SQP(K/R)^19 ^and ^46 ^SYGR^49 ^sequences of Tat were conserved among different HIV-1 isolates and whether mutations in these residues correlated with apparent progression of disease in the patient from whom the isolates were obtained. In this investigation, we studied 105 sequences of Tat deposited to PubMed database [34]. Of these, 55 were obtained from patients who were not on antiretroviral treatment and were classified as being healthy, and 50 were from patients who were classified as being ill and who were on antiretroviral treatment. We found that R^49 ^was absolutely conserved, and thus no correlation could be obtained. In contrast, Table 2 shows that 29 (53%) of the "healthy" HIV patients had a (K/R)^19 ^mutation compared to only 4 (8%) of the "ill" HIV patients (Pearson Chi-square correlation 24.312, df = 1; P < 0.001). This result suggests physiological importance of mutation of position 19 of Tat for progression of HIV-1 disease, and are consistent with the possibility that ^16^SQP(K/R)^19 ^sequence is a putative CDK2 recognition site in Tat.

**Table 2:** Mutation of (K/R)^19 ^residue sequence of Tat and Sickness Status

	***Healthy ***	***Sick ***	**Total**
***Non-mutated Tat (K/R)19***	26	46	72
***Tat (K/R)19(T, A or G)***	29	4	33
***Total***	55	50	105

## Discussion

Our recent studies indicate that Tat's role in HIV-1 transcription is extremely complex and may not confine solely to the interaction with CDK9/cyclin T1. We have previously reported that Tat interacts directly or indirectly with host cell protein phosphatase 1 (PP1) and protein phosphatase 2A (PP2A). Tat binds to protein phosphatase-1 (PP1) and this binding is important for the induction of HIV-1 transcription by Tat [31,35]. Tat interaction with PP1 is intriguing as PP1 regulates CDK9 phosphorylation *in vivo *[36]. We recently showed that Tat binds to LIS1 protein, a product of lissencephaly gene which mutations cause a severe brain malformation [37]. LIS1 resembles the B-subunit of PP2A and interacts with the catalytic subunit of PP2A; and LIS1 expression induces HIV-1 transcription [38]. Thus Tat might also be able to interact with PP2A, although not directly with its catalytic subunit. The current study adds more complexity to the Tat function showing that Tat might undergo phosphorylation by CDK2/cyclin E.

We hypothesized here that CDK2 affects HIV-1 transcription by phosphorylating Tat and that Tat phosphorylation might be important for HIV-1 transcription. We show here that Tat undergoes phosphorylation *in vivo *on serine residues, and that CDK2 is involved in this phosphorylation. Our findings also indicate that phosphorylation of Tat is important for HIV-1 transcription and the activation of integrated HIV-1 provirus. In our previous work we demonstrated that CDK2/cyclin E phosphorylates the C-terminal domain of RNA polymerase II *in vitro *[39-41]; that CDK2 was required for Tat-dependent transcription *in vitro*, and that CDK2 phosphorylates HIV-1 Tat *in vitro *[20,41]. CYC202 (R-roscovitine), a pharmacological inhibitor of CDK2, efficiently inhibited replication of wild type and HAART resistant HIV-1 mutants in T-cells, monocytes and PBMCs [42] indicating that CDK2 activity is required for HIV-1 replication. Recently we showed that siRNA-directed against CDK2 inhibits Tat-induced HIV-1 transcription and HIV-1 viral replication [33]. Thus our present study as well as our previous studies point to CDK2 as an important regulator of HIV-1 transcription. Until recently CDK2/cyclin E was considered to be essential for cell cycle progression and that CDK2 regulates G1/S transition by phosphorylating Rb-sequestering factors, including E2F [43]. Recent findings challenged this role of CDK2. CDK2 knock-out mice were viable [44], suggesting that CDK2 is dispensable for proliferation and survival of most cell types. Also, inhibition of CDK2 activity through expression of p27 Kip1, dominant-negative CDK2, antisense oligonucleotides or siRNA did not have an effect on growth of several tumor cell lines [45]. Therefore, not being essential for cellular viability, CDK2 might present a feasible target for anti-HIV-1 therapeutics.

Previous attempts to detect Tat phosphorylation *in vivo *were not successful [25]. It is possible that low level of Tat expression or fast dephosphorylation in the cells or during sample preparation may not allow easy detection of Tat phosphorylation. For example, in the early studies Ben Berkhout and his colleagues could only detect Tat expression in COS-7 cells but not in HeLa cells [46-48]. We found that expression of Flag-tagged Tat allowed higher levels of Tat expression especially with the adeno-virus mediated delivery. Treatment with okadaic acid, which inhibits phosphatases of the PPP-family including PP1 and PP2A [49], significantly enhanced Tat phosphorylation (Fig. 3), suggesting that Tat may be dynamically dephosphorylated by a cellular PPP-type phosphatase. When we inhibited PP1 by over expression of the central domain of nuclear inhibitor of PP1 (NIPP1) [31] we did not detect changes in Tat phosphorylation (data not shown). Thus PP2A rather than PP1 is a candidate phosphatase to dephosphorylate Tat.

Our analysis showed that inhibition of CDK2 expression by siRNA substantially blocked Tat phosphorylation and prevented association of Tat with CDK2. Although these findings suggest that CDK2 might directly phosphorylate Tat, we also cannot rule out a possibility that inhibition of CDK2 reduces the activity of another kinase that in turn might be involved in Tat phosphorylation. Finding that Tat co-migrates with CDK2 on glycerol gradient and also co-precipitated with CDK2 confirms our previous observation that Tat-associated kinase activity contained CDK2 [41]. CDK2-directed siRNA significantly reduced association of CDK2 to Tat, probably by reducing the amount of CDK2 available to interact with Tat.

We found that Tat is phosphorylated on serine residues *in vivo*. We previously suggested that the ^16^SQPK^19 ^and K^41 ^× L^43 ^sequences of Tat interact with CDK2 and cyclin E respectively, and that S16 is phosphorylated by CDK2 [20]. In the present study we found that both S^16 ^and S^46 ^of Tat are potential phosphorylation sites. As S^46 ^is adjacent to the K^41 ^× L^43 ^sequence of Tat, it is likely that the K^41 ^× L^43 ^sequence participates in binding to CDK2 rather than to cyclin E, as we originally suggested. Interestingly, recombinant CDK2/cyclin E only phosphorylated full length Tat 1–72 but not the 15 amino acid peptides of Tat, ^9^EPWKHPGSQPKTACN^23 ^or ^37^CFTTKGLGISYGRKK^51 ^(AIDS Research and Reference Reagents Program, NIH), containing only the phosphorylation sites (data not shown), which may indicate a requirement of additional sequences of Tat for its interaction with CDK2/cycline E. Another explanation is that full length Tat creates a favorable conformation for phosphorylation by CDK2. The sequences ^16^SQP(K/R)^19 ^and ^46^SYGR^49 ^only partially match the CDK2 (S/T)_0_P_1_K_2_(K/R)_3 _phosphorylation motif. Although the catalytic efficiency of CDK2-cyclin A is impaired 2000-fold, when Pro_1 _is substituted with Ala in a short synthetic peptide substrate, physiological substrates for both CDK2-cyclin A and CDK2-cyclin E often contain phosphorylation motifs replaced with sub optimal determinants [28]. In such sub optimal substrates phosphorylation is enhanced by a cyclin-binding motif that compensates for otherwise poor catalysis [28]. Therefore, binding of cyclin E still might be important for efficient phosphorylation of Tat by CDK2.

Using mutation analysis, we found that S^16 ^and S^46 ^are equally important for activation of integrated proviral DNA. The single point mutants did not show a significant level of activation, and the double mutant Tat was completely inactive in HLM-1 cells. In contrast, mutation of S^16 ^and S^46 ^moderately reduced activation of HIV-1 transcription from the episomal promoter. Thus Tat S^16 ^and S^46 ^residues are important for transcription of a full genomic HIV-1 template containing natural chromatin structure. The effect of alanine mutation of S^46 ^is at variance with the previously published observation that alanine mutation of S^46 ^induces Tat-transactivation [50]. We did not see an increase of Tat transactivation with all mutants tested. Thus we cannot explain this discrepancy. Tat was proposed to form aggregates in the nucleus [51]. Using yeast two-hybrid system, we found no evidence that Tat forms dimers in yeast cells (not shown). Therefore, it remains to be determined why Tat should undergo phosphorylation to be fully active as a transcriptional activator. We did not detect a difference between WT and mutant Tat in ability to bind to TAR RNA (not shown). We observed an increase in the expression of untagged Tat with mutations in the S^16 ^or Ser^46 ^Tat residues and particular of the double mutant of Tat (Fig. 8A). An increase in Tat expression was observed earlier by Rice and Carlotti with a mutant of Tat that lacked first 36 N-terminal amino acids [50]. Thus the amount of Tat expressed in the cells might be stringently controlled and the excess of Tat might have a negative effect on Tat transactivation. Another possibility is that Tat may need to undergo non-proteolytic ubiquitination by Hdm2 ubiquitin ligase [22] to be fully active as a transactivator. It is possible that Tat phosphorylation may facilitate ubiquitination of Tat by Hdm2 similar to phosphorylation-dependent ubiquitination of p53 by Hdm2 [52].

Analysis of Tat sequences available in the PubMed showed that Tat isolates contain from 4 to 11 serine residues. In addition to the highly conserved S^16 ^and S^46 ^residues, Tat contains less conserved serines at positions 23, 61, 62, 68, 70, 73, 74 and 75. Analysis of the ^16^SQP(K/R)^19 ^and ^46^SYGR^49 ^sequences of Tat, showed that R^49 ^is conserved among different HIV-1 isolates. Interestingly, mutations in the (K/R)^19 ^residue showed correlation with non-progression of HIV-1 disease. Thus (K/R)^19 ^residue which is part of a putative CDK2 recognition site in Tat, may be important for progression of HIV-1 disease. Future study will address whether mutation of (K/R)^19 ^residue is important for phosphorylation of Tat by CDK2 and whether mutations in this residue affect viral replication.

Taken together, our findings indicate that Tat is phosphorylated *in vivo *and that phosphorylation of Tat is important for the activation of integrated HIV-1 provirus. Our finding also indicates that CDK2 associates with Tat and thus is likely to phosphorylate Tat directly *in vivo*. Our findings open the door to the evaluation of the potential efficiency of presently available CDK2 inhibitors and specifically designed future inhibitors to disrupt CDK2-Tat association.

## Methods

### Materials

293T cells and COS-7 cells were purchased from ATCC (Manassas, VA). HeLa-MAGI cells [53], HLM-1 cells [54], anti-Tat rabbit polyclonal (HIV-1 BH10 Tat antiserum, [55]) and monoclonal (NT3 2D1.1, courtesy of Dr. Jonathan Karn) antibodies were received from the AIDS Research and Reference Reagents Program (NIH). Anti-Flag monoclonal antibodies, anti-α-tubulin antibodies, protein (G) and protein (A) agarose and okadaic acid were purchased from Sigma (Atlanta, GA). All radioactive reagents were purchased from GE Health Care Life Sciences. The 3,3'-diaminobenzidine enhanced liquid substrate system for membrane ELISA (DABM) was purchased from Sigma (St Louis, MO). Antibodies for CDK2, CDK9, cyclin T1 and cyclin T2 were purchased from Santa Cruz Biotechnology (Santa Cruz, CA). Antibodies against RNAPII were from Babco. Anti-Brd4 and anti-HEXIM1 antibodies were a gift from Dr.Q. Zhou (University of California, Berkeley). HIV-1 Tat was expressed in *Escherichia coli *and purified on Aquapore RP-300 column (Applied Biosystems, Foster City, CA) by reverse-phase chromatography as we described [20].

### Plasmids

Tat expression plasmid was a gift from Dr. Ben Berkhout (University of Amsterdam) [48]. The Flag-Tat was cloned into the adeno-CMV-link vector as described below and verified by sequencing. The S16A and S46A mutations of the sequence of Tat were made according to the Quick-Change site-directed mutagenesis protocol of Stratagene, using the appropriate primers and templates. The sequences of the DNA constructs were verified by sequencing using a commercial service from Macrogen (Seoul, Korea).

### CDK2/cyclin E purification

CDK2 and cyclin E were purified from lysates of Sf9 insect cells infected with baculoviruses producing CDK2 and cyclin E. Proteins were purified essentially as described earlier [56]. Briefly, 1 ml of cells (from 250 ml of culture) was lysed with 16 ml of lysis buffer (50 mM Tris-HCl, pH 8.0, 10 mM 2-mercaptoethanol, 10% glycerol and with PMSF), homogenized on ice and centrifuged at 45,000 g for 1 hr at 4°C. Supernatant was loaded on Mono-Q 10/10 column (Amersham, USA). Two separate cell cultures, one infected with CDK2-expressing baculovirus and the other one infected with cyclin E-expressing baculovirus were used for purification. The Mono-Q fractions containing CDK2 or cyclin E were mixed 1:1 and loaded onto Superdex column (Sephadex H200, Amersham, USA). Purity of CDK2/cyclin E was checked on 12% PAGE followed by Coomassie staining (Additional file 1). We also analyzed the Superdex fractions by immunoblotting with andti-CDK2 antibodes and assayed their enzymatic activity using histone H1 and purified Tat proteins as substrates. Fractions containing CDK2/cyclin E were concentrated using Microcon tubes (Amicon, USA).

### In vitro kinase assay

CDK2 kinase assays were performed at 30°C for 30 min in kinase assay buffer (50 mM HEPES-KOH, pH 7.9, 10 mM MgCl_2_, 6 mM EGTA, 2.5 mM DTT) containing histone H1 or purified Tat protein, 200 μM ATP and (γ-P^32^)ATP. A mixture was incubated for 30 min at 30°C, reaction was stopped with 8 μl of 4× SDS buffer and resolved on 12% PAGE.

### Immunoprecipitation of CDK2 and kinase assay with Tat

293T cells were transfected with CDK2-directed or non-targeting siRNA. At 48 hours after transfection cells were lysed in whole cell lysis buffer. About 50 μg of protein lysate was subjected to precipitation with anti-CDK2 rabbit antibodies (600 ng/IP) on 40 μl protein A agarose beads (50% slurry) (Sigma). As a control, rabbit preimmune serum was used. Precipitation was carried out for 2 hrs at 4°C. The beads were washed with TNN buffer, then with TTK buffer and supplemented with 20 μl of kinase mixture containing TTK buffer, 100 μM ATP, 0.5 μCi of γ-(^32^P)ATP and 1 μg of purified Tat. The kinase reaction was carried out for 15 min at 30°C, reaction was stopped with 8 μl of 4× SDS-loading buffer and resolved on 12% Tris-Tricine gel. The gel was stained with Coomassie blue, dried and exposed to Phosphor Imager screen.

### Fractionation of cellular lysates on glycerol gradient

293T cells were infected with Adeno-Tat virus with MOI 1, and incubated overnight. The cells in 100 mm plate were lysed with 0.5 ml of whole cell lysis buffer (50 mM Tris-HCl, pH 7.5, 0.5 M NaCl, 1% NP-40, 0.1% SDS) supplemented with protease cocktail (Sigma) and RNasin (Amersham). Cell lysates were clarified by centrifugation for 30 min at 10,000 g and loaded on top of 10% to 30% glycerol (9 ml) gradient. Glycerol gradient buffer contained 20 mM HEPES-KOH, pH 7.9, 150 mM KCl, 200 μM EDTA

The gradient was spun in SW 41Ti rotor (Beckman) at 38,000 rpm for 18 hours. Fractions (0.5 ml) were collected through a needle inserted to the bottom of the tube and analyzed by Immunoblotting.

### Tat trypsinization and chromatographic separation of peptides

Non-phosphorylated and phosphorylated Tat were resolved by 15% Tris-Tricine SDS-PAGE. A gel piece containing Tat was crushed and incubated with 1 μg of porcine trypsin (Promega, Madison, WI) in 0.1 M NaHCO_3 _(pH 7.9) overnight at 37°C. The extracted peptides were lyophilized, dissolved in 0.1% TCA and separated by reverse-phase chromatography on a μRPC C2/C18 ST 4.6/100 column (Amersham Pharmacia Biotech) using AKTA purifier (AmershamPharmaciaBiotech). Eluent A was 0.1% TCA in water and eluent B was 0.1% TCA in 90% acetonitrile. The gradient was 5% B for 2 column volumes (CVs), 5–50% B for 20 CVs, 50–100% B for 12 CVs and 100% B for 4 CVs. The flow rate was 0.5 ml/min.

### MALDI-TOF mass spectrometry and peptide sequencing

Fractions from the HPLC separation described above were lyophilized. Multiple peptide sequences were determined in a single run by Applied Biosystems Maldi-TOF/TOF 4700 proteomics analyzer (25–30,000 resolution in reflectron mode, 5 ppm accuracy with IS, subfmole sensitivity for peptide mass fingerprints (PMF), fmole sensitivity for PSD/CID capacity of >36,000 PMF per 24 hours). Data were analyzed utilizing a local multi-processor installation of MASCOT MS PMF and MS/MS PSD, CID peptide identification software. GPS also supports an integrated MS-MS/MS mode and a chromatographic separation mode with SEQUEST-like capability.

### Preparation of Adeno-Tat

The E1-deleted recombinant Ad carrying Tat was generated as previously described [31]. Briefly, a cDNA fragment encoding the full length HIV-1 Tat protein was cloned into the plasmid pCXN was subcloned in the pAd.CMV link plasmid. The Tat-coding DNA corresponds to the HIV-1 isolate P9.3 from United Kingdom (Accession number AF324447). The DNA sequence is ATGGACTACAAGGACGACGA TGACAAAGAA TTCATGGAGC CAGTAGATCCTAGACTAGAG CCCTGGGAGC ATCCAGGAAG TCAGCCTAAG ACTGCTTGTACCCCTTGCTA TTGTAAAAAG TGTTGCTTTC ATTGCCAAGT TTGTTTCACAACAAAAGGCT TAGGCATCTC CTATGGCAGG AAGAAGCGGA GACAGCGACGAAGAGCTCCT CAAGACAGTC AGACTCATCA GGCTTCTCTA TCAAAGCAATCCCTACCCCA AACCCAGAGG GACTCGACAG GCCCGGAAGA ATCGAAGAAGGAGGTGGAGA GCAAGGCAGAGACAGATCGA TTCGATTA. It corresponds to the protein sequence of Tat containing Flag epitope at its N-terminus-MDYKDDDDKEFMEPVDPRLEPWEHPGSQPKTACTPCYCKKCCFHCQVCFTTKGLGISYGRKKRRQRRRAPQDSQTHQASLSKQSLPQTQRDSTGPEESKKEVESKAETDRFD. pAd.CMVlink and Cla I digested adenoviral DNA were co-transfected into HEK293 cells at a ratio of 3:1 by calcium phosphate precipitation to allow the recombination. The virus was purified through three rounds of plaque-purification. Viruses were replicated in HEK293 cells and were purified from a cell lysate by two rounds of CsCl density gradient centrifugation. The purified virus was desalted on a Bio-Gel P-6 desalting column (Bio-Rad Laboratories, Hercules, CA) equilibrated with PBS. The titer of the virus preparation was determined both by absorbency at 260 nm and by plaque assay. The particle to plaque forming unit ratio was less than 100. Purified viruses were suspended in PBS at the desired concentrations.

### Tat phosphorylation in vivo

HeLa cells were infected with recombinant Adenovirus carrying Flag-tagged Tat prepared as we described [31]. The purified virus had a particle to plaque forming unit (Pfu) ratio of less than 100. We added approximately 10 Pfu per cell to achieve high level of Tat expression in infected HeLa cells. At 48 hours post infection the media was changed for 1 hour to a phosphate-free DMEM media (Life Technologies, Rockville, MD) containing no serum. Then the media was changed to phosphate-free DMEM supplemented with 0.5 mCi/ml of (^32^P)-orthophosphate and cells were further incubated for 2 hours at 37°C. Where indicated, 1 μM okadaic acid (Sigma) was added to block cellular PPP-phosphatases. Cells were washed with PBS and lysed in whole cell lysis buffer (50 mM Tris-HCl, pH 7.5, 0.5 M NaCl, 1% NP-40, 0.1% SDS) supplemented with protease cocktail (Sigma). After 10 min on ice, cellular material was scraped and then centrifuged at 14,000 rpm, 4°C for 30 min. The supernatant was recovered and immediately used for immunoprecipitation. Tat was precipitated with anti-Flag monoclonal antibodies coupled to protein G agarose and with polyclonal anti-Tat antibodies coupled to protein A agarose for 2 h at 4°C in a TNN Buffer containing 50 mM Tris-HCl, pH 7.5, 0.15 M NaCl, and 1% NP-40. The immunoprecipitated Tat was recovered by heating for 2 min at 100°C in Tricine SDS-loading buffer, resolved on 15% Tris-Tricine SDS-PAGE [32] and transferred to polyvinylidene fluoride (PVDF) membranes (Millipore, Allen, TX). The membrane was analyzed with anti-Tat monoclonal antibodies using 3,3'-Diaminobenzidine enhancer system (Sigma) and was also exposed to Phosphor Imager screen (Packard Instruments, Wellesley, MA).

### Phosphoamino acid analysis

The analysis was carried out on the Hunter thin layer peptide mapping electrophoresis system (CBS, Del Mar, CA) according to the manufacturer's recommendations. Briefly, phosphorylated Tat, prepared as described above was resolved on 15% SDS-Tris-Tricine PAGE and the gel was dried on a Whatman paper. The portion of the gel containing Tat was excised, rehydrated and treated overnight with trypsin to elute Tat. The eluted peptides were boiled in 5.7 M HCl at 110°C to liberate (^32^P)-labeled phospho-amino acids as described [57]. After the hydrolysis, the sample was lyophilized and resuspended in the buffer for pH 1.9 electrophoresis also containing the phosphoamino acid standards at 0.06 mg/ml. The sample was resolved by thin-layer electrophoresis on a cellulose plate (CBS, Del Mar, CA) at pH 1.9 in the first direction and at pH 3.5 in the second direction. Cold standards were visualized by staining the plate with 0.25% ninhydrine dissolved in ethanol. Positions of labeled phosphoamino acids were analyzed with Phosphor Imager (Packard Instruments, Wellesley, MA).

### Transfection and HIV-1 detection from HLM1 cells

HLM-1cells were derived from HeLa-T4+ cells integrated with one copy of HIV-1 genome containing a Tat-defective mutation. The mutation was introduced as a triple termination linker (TTL) at the first AUG of Tat gene [54]. HLM-1 cells are negative for virus particle production HLM1 cells are negative for virus particle production. HLM1 cells can be induced to express non-infectious HIV-1 particles after transfection with Tat cDNA, or by treatment with mitogens such as TNF-α or sodium butyrate. HLM1 cells were grown in DMEM media containing 100 μg/ml of G418, plus 1% streptomycin, penicillin antibiotics and 1% L-Glutamine (Gibco/BRL). The cells grown up to 75% confluence were transfected with Tat expression vectors, including wild type Tat, S16A, S46A, and S16A/S46A mutant Tat plasmids using the calcium phosphate method. The transfected cells were washed after four hrs and fresh complete DMEM media with 10% fetal bovine was added for the remainder of the experiment. The p24 gag antigen was detected in the supernatants of transfected cells using a standard ELISA kit (Abbott).

### siRNA treatment

CDK2-directed siRNA pool (M-003236-03-005) and negative control pool (D-001206-13-05) were purchased from Dharmacon (Dallas, TX). The siRNAs were transfected at final concentration of 100 nM using Lipofectamin reagent (Invitrogen) according to the manufacturer's recommendations. The siRNAs were incubated with cells for 2 days before cells were labeled with ^32^P or lysed for Western blotting analysis.

## Competing interests

The author(s) declare that they have no competing interests.

## Authors' contributions

TA purified recombinant CDK2/cyclin E, performed *in vitro *Tat phosphorylation, carried out the experiments with the CDK2-directed siRNA, CDK2-Tat precipitation experiments, Western blot analysis of glycerol gradients and also helped with *in vivo *(^32^P) phosphorylation experiments and with Tat mutagenesis. RB and ZK carried out experiments with live virus. MJ prepared adeno-Tat virus, helped to analyze the expression of Tat in cultured cells and prepared samples for MALDI-TOF analysis. MJ and SN were also running glycerol gradients. SC performed statistical analysis of the HIV-1 isolates. EE performed MALDI TOF analysis of Tat peptides. WS, VRG and FK participated in the design and discussion of the study. SN performed *in vivo *Tat phosphorylation experiments, phosphoaminoacid analysis of Tat and Tat mutagenesis, and also performed general control and coordination of the study. All authors read and approved the manuscript.

## Supplementary Material

Additional File 1 Purification of CDK2/cyclin E. Mixed mono Q fractions of CDK2 and cyclin E (Mono Q lane) were purified on Superdex column. Fractions 37, 39, 41, 43, and 45 were analyzed for the presence of CDK2 and cyclin E by Coumassie staining. Click here for file
