# Supplementary figures and images for: Phosphorylation of HIV-1 Tat by CDK2 in HIV-1 transcription
Source: Retrovirology. 2006 Nov 3;3:78. doi: 10.1186/1742-4690-3-78 (PMC1636661; doi:10.1186/1742-4690-3-78)

MW MQ 37 39 41 43 45

kDa

70 -

60 -

50 -

40 -

30 -

25 -

20 -

15 -

10 -

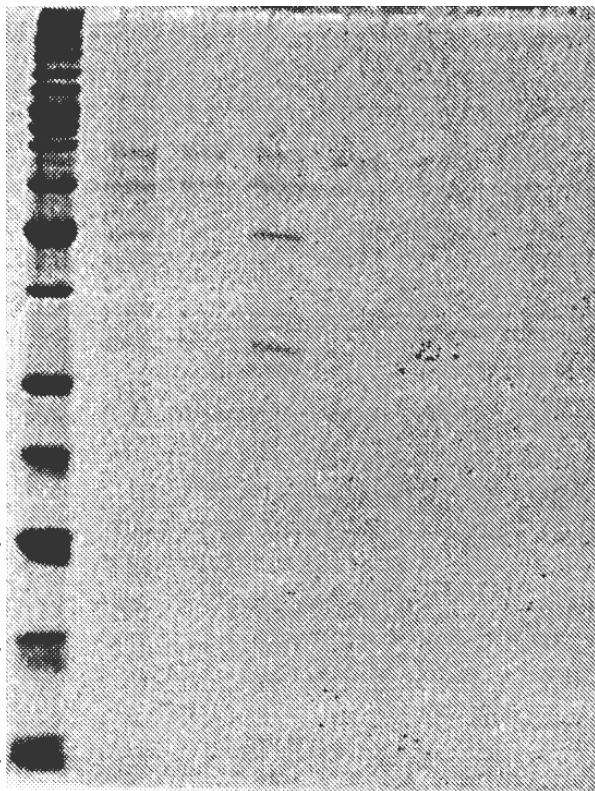

◀ Cyclin E

◀ CDK2

Supplement: Additional File 1 — Purification of CDK2/cyclin E. Mixed mono Q fractions of CDK2 and cyclin E (Mono Q lane) were purified on Superdex column. Fractions 37, 39, 41, 43, and 45 were analyzed for the presence of CDK2 and cyclin E by Coumassie staining. [file 1742-4690-3-78-S1.pdf]
